# Ultrastrong conductive in situ composite composed of nanodiamond incoherently embedded in disordered multilayer graphene

**DOI:** 10.1038/s41563-022-01425-9

**Published:** 2022-12-15

**Authors:** Zihe Li, Yujia Wang, Mengdong Ma, Huachun Ma, Wentao Hu, Xiang Zhang, Zewen Zhuge, Shuangshuang Zhang, Kun Luo, Yufei Gao, Lei Sun, Alexander V. Soldatov, Yingju Wu, Bing Liu, Baozhong Li, Pan Ying, Yang Zhang, Bo Xu, Julong He, Dongli Yu, Zhongyuan Liu, Zhisheng Zhao, Yuanzheng Yue, Yongjun Tian, Xiaoyan Li

**Affiliations:** 1 Center for High Pressure Science (CHiPS), State Key Laboratory of Metastable Materials Science and Technology, Yanshan University, Qinhuangdao, China; 2 Center for Advanced Mechanics and Materials, Applied Mechanics Laboratory, Department of Engineering Mechanics, Tsinghua University, Beijing, China; 3 Key Laboratory of Microstructural Material Physics of Hebei Province, School of Science, Yanshan University, Qinhuangdao, China; 4 Department of Chemistry and Bioscience, Aalborg University, Aalborg, Denmark

**Keywords:** Mechanical properties, Nanoscale materials

## Abstract

Traditional ceramics or metals cannot simultaneously achieve ultrahigh strength and high electrical conductivity. The elemental carbon can form a variety of allotropes with entirely different physical properties, providing versatility for tuning mechanical and electrical properties in a wide range. Here, by precisely controlling the extent of transformation of amorphous carbon into diamond within a narrow temperature–pressure range, we synthesize an in situ composite consisting of ultrafine nanodiamond homogeneously dispersed in disordered multilayer graphene with incoherent interfaces, which demonstrates a Knoop hardness of up to ~53 GPa, a compressive strength of up to ~54 GPa and an electrical conductivity of 670–1,240 S m^–1^ at room temperature. With atomically resolving interface structures and molecular dynamics simulations, we reveal that amorphous carbon transforms into diamond through a nucleation process via a local rearrangement of carbon atoms and diffusion-driven growth, different from the transformation of graphite into diamond. The complex bonding between the diamond-like and graphite-like components greatly improves the mechanical properties of the composite. This superhard, ultrastrong, conductive elemental carbon composite has comprehensive properties that are superior to those of the known conductive ceramics and C/C composites. The intermediate hybridization state at the interfaces also provides insights into the amorphous-to-crystalline phase transition of carbon.

## Abstract (web summary)

In situ composites consisting of nanodiamond homogeneously dispersed in disordered multilayer graphene with incoherent interfaces and complex bonding are synthesized that exhibit an ultrahigh hardness and compressive strength, and excellent electrical conductivity.

## Main

High-performance materials that couple high strength/hardness and electrical conductivity are in demand for a broad range of applications. Traditional metals have excellent conductivity, but their yield strength is generally lower than 2 GPa, and they become soft at relatively high temperatures, compared to most ceramics and carbon materials^1^. Ceramics generally possess superior strength/hardness, wear resistance and high-temperature stability, but most of them are good electrical insulators^2^. Ceramics can be made conductive by doping^3^ or adding conductive second phases including metals and carbon materials such as graphene, nanotubes and nanofibres^4,5^. However, due to the low diffusivity of dopants in ceramics, the doping concentration is limited^3^. Compared with single-phase ceramics, conductive ceramic composites exhibit lower strength, lower hardness and lower scratch resistance, as well as lower thermal stability owing to the weak hetero-interface between the matrix and the second phase.

The uniqueness of elemental carbon lies in its flexibility to form *sp*, *sp*^2^ and *sp*^3^ bonds, resulting in the formation of a variety of allotropes from soft, conductive graphite to superhard, insulating diamond. The carbon forms with mixed hybridization states are expected to integrate the advantages of each single hybrid state and possess versatile mechanical and electrical properties. Various *sp*^2^–*sp*^3^ mixed amorphous carbon materials have been prepared by multiple deposition techniques from carbonaceous precursors^6^ or by a pressure-induced phase transition of *sp*^2^ carbon materials such as fullerenes and glassy carbon (GC)^7^. The fullerene C_60_ undergoes crystal-to-amorphous and amorphous-to-amorphous transitions when heated during compression and transforms into C_60_ polymers with different dimensionalities as well as distinct amorphous phases before transforming into diamond^8,9^. Likewise, GC undergoes amorphous-to-amorphous and amorphous-to-diamond transitions under different pressure and temperature conditions^10–15^. This is because carbon has a complex energy landscape, and metastable phases with local energy minima may be formed due to a preferable kinetic transformation. Therefore, unique metastable phases or multiphase composites are expected to be obtained by controlling the phase transition of high-energy precursors by varying temperature and pressure^8–10^.

Direct combination of two or more carbon materials is another strategy to generate superior material properties. Traditional C/C composites, such as carbon-fibre-reinforced pyrolytic carbon, are made of *sp*^2^-hybridized carbon materials with a variety of microstructures, from disordered, poorly graphitic fragments to oriented, highly graphitized crystallites, and they have been widely used in space aircraft, the automobile industry and biomedical devices^16,17^. These C/C composites possess high tensile strength (200–350 MPa) and electrical conductivity (2.0–5.9 × 10^5^ S m^–1^)^16,17^, but further improvement of their mechanical performance becomes almost impossible because of the weak van der Waals bonding within/between components. By introducing ultrastrong components into C/C composites to realize strong covalent bonding between the component interfaces, the comprehensive mechanical properties would be greatly improved. However, this is not feasible, since it is difficult to chemically create a strong interface connection between diamond and other types of carbon materials.

Here we study the transformation of GC into diamond under high temperature and high pressure, and find that the transformation is a nucleation process of diamond through local rearrangement of carbon atoms towards lower potential energy. This process differs from the transformation of graphite into diamond^18^. A unique C/C composite was synthesized by controlling the amorphous-to-crystalline transition in a narrow temperature range under pressure. The composite is composed of a disordered multilayer graphene matrix and nanodiamond, and the two phases are interconnected mainly through an incoherent interface. Note that ‘incoherent interface’ refers to an interface where the two phases are irregularly and non-uniformly connected. Such a unique phase composition and interface enable the composite of nanodiamond and disordered, multilayer graphene (ND/DMG) to achieve a combination of ultrahigh hardness and strength and excellent electrical conductivity.

## Results

### Microstructure

**Fig. 1:**
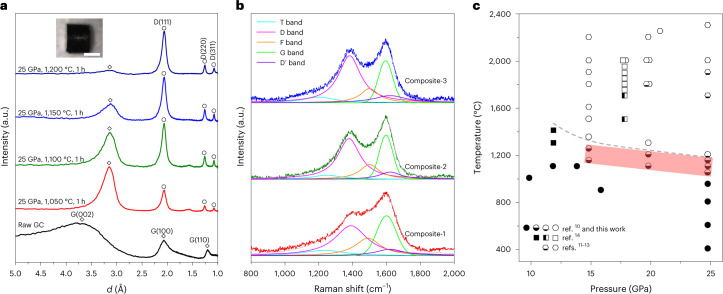
X-ray diffraction patterns and Raman spectra of ND/DMG composite and *P*–*T* phase diagram of glassy carbon. **a**, X-ray diffraction patterns measured under ambient conditions. D represents the diffraction peaks of diamond, and G represents the diffraction peaks of disordered multilayer graphene. The inset shows the morphology of the recovered sample rod. Scale bar, 500 μm. *d*, interplanar spacing. **b**, Raman spectra measured at ambient conditions. In **a** and **b**, Composite-1, Composite-2 and Composite-3 represent the specimens recovered after compressing GC samples at 25 GPa and temperatures of 1,050, 1,100 and 1,150 °C, respectively. In **b**, the green, magenta, orange, cyan and violet peaks represent the Raman vibration of the G band, D band, F band, T band and D′ band, respectively. **c**, The *P*–*T* phase diagram of GC. Solid symbols represent compressed GC^10^ or unchanged GC micro-balls^14^; half-filled circles represent the ND/DMG composite; hollow symbols represent pure NPD or NCD^11–14^; minor-segment-filled symbols represent almost-pure diamond samples with a small amount of ‘compressed graphite’^13^; and half-filled squares represent the products with NCD micro-balls and unchanged GC micro-balls together after high-pressure and high-temperature treatment^14^. The shaded region indicates the *P* and *T* conditions for forming a ND/DMG composite with good electrical conductivity. Below the grey dashed curve is the area where the synthesized samples have good conductivity.

**Extended Data Fig. 1:**
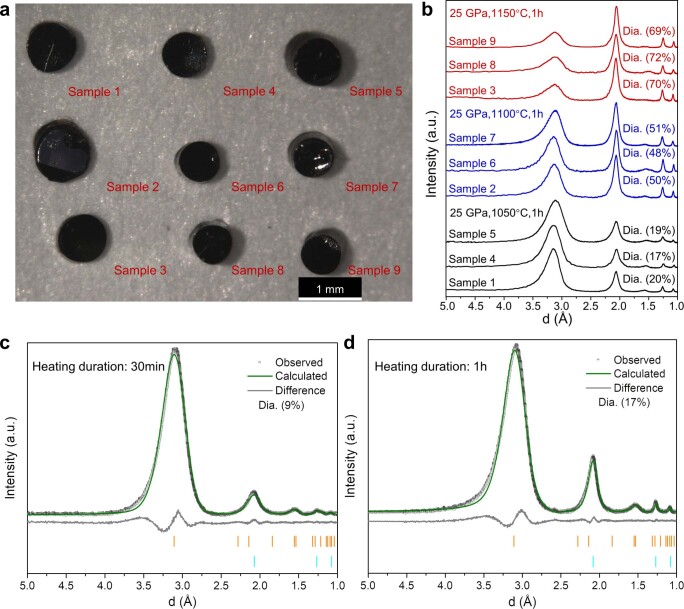
Optical images (a), XRD patterns (b) of ND/DMG composite samples synthesized at nominal synthesis temperatures (that is 1050˚C, 1100˚C and 1150˚C) under pressure of 25 GPa with heating duration of 1 h and Rietveld refinement analysis of samples recovered from 25 GPa and 1050 °C with heating durations of 30 min (c) and 1 h (d). Sample1, Sample 4 and Sample 5 are recovered from 25 GPa, 1050 °C; Sample 2, Sample 6 and Sample 7 are recovered from 25 GPa, 1100 °C; Sample 3, Sample 8 and Sample 9 are recovered from 25 GPa, 1150 °C. Sample 1, 2 and 3 correspond to the Composite-1, Composite-2 and Composite-3 in the main text, respectively. The contents of diamond and disordered multi-layer graphene in the composite were obtained from Rietveld refinement. The difference of diamond content in the samples obtained at each synthesis temperature is no more than 3%. Cyan bars: diamond diffraction peak positions, orange bars: diffraction peak positions of graphite with a reduced interlayer spacing. With the increase of heating duration from 30 min to 1 h, the content of diamond in the composite increases from 9% to 17%.

Figure 1a shows the X-ray diffraction patterns of samples recovered from the compression of GC at a pressure of 25 GPa and temperatures from 1,050 °C to 1,150 °C for 1 hour. The selection of experimental conditions is described in Supplementary Text 1. The recovered samples have four main diffraction peaks around 3.12, 2.06, 1.26 and 1.08 Å. The first broad peak arises from the interlayer spacing of disordered graphene fragments (G) in the compressed GC, and the mean interlayer spacing is shorter than that of raw GC (~3.62 Å) due to the randomly distributed *sp*^3^ nodes between graphene layers^10^. The last three wide peaks can be attributed to the {111}, {220} and {311} diffraction of cubic diamond consisting of fine grains (D). The samples quenched from 1,050 °C, 1,100 °C and 1,150 °C are depicted as Composite-1, Composite-2 and Composite-3, respectively. With the increase of synthesis temperature, the diffraction peak at ~3.12 Å from the disordered graphene interlayer is gradually weakened compared to those of diamond, indicating the increased diamond content in the composite. The volume percentages of diamond in Composite-1, -2 and -3 are determined as ~20%, 50% and 70% via the Rietveld refinement method, respectively. We also prepared more samples at each nominal synthesis temperature (that is, 1,050 °C, 1,100 °C and 1,150 °C) at 25 GPa. The corresponding optical images and X-ray diffraction patterns of the samples are shown in Extended Data Fig. 1. The difference in the diamond content in the composite samples obtained at each synthesis temperature is no more than 3%. To study the effect of the heating duration on the phase fraction, the syntheses of samples were carried out at 25 GPa and 1,050 °C for 30 min and 1 h, respectively. The X-ray diffraction patterns of two synthetic samples are also shown in Extended Data Fig. 1. With the increase of heating duration from 30 min to 1 h, the content of diamond in the composite increases from 9% to 17%, indicating that the heating duration indeed affects the phase fraction in the samples.

As shown in Fig. 1b, the Raman features of the composite resemble those found in natural and synthetic diamond-related materials^19^. Recent studies indicate that such Raman features can be associated with proposed type 2 diaphite structures^19^. To estimate the graphene-like cluster sizes in the composite, the Raman spectra of the composite are fitted and deconvoluted into the G band at ~1,570–1,600 cm^−1^, D band at ~1,380 cm^−1^, F band at ~1,450–1,470 cm^−1^, T band at ~1,100–1,200 cm^−1^ and D′ band at ~1,620 cm^−1^ (ref. ^9^). The G band is associated with the in-plane stretching vibration of various *sp*^2^-bonded structures, while the T band arises from the *sp*^3^-bonded features and the D′ band, from double-resonant, defect-related graphene features. The intensities of the D and F bands reflect the number of hexagonal and pentagonal aromatic rings in small clusters, respectively. With the increase of synthesis temperature, the D band becomes stronger, whereas the G and F bands get weaker. This indicates the gradual growth of the number of hexagonal rings in the system at the expense of other *sp*^2^ carbon structural units. The graphene-like cluster sizes (*L*_a_) of Composite-1, Composite-2 and Composite-3 are estimated from the area ratio between the D and G bands^9^ and thereby are found to be ~6.5, 5.8 and 4.7 nm, respectively.

**Extended Data Fig. 2:**
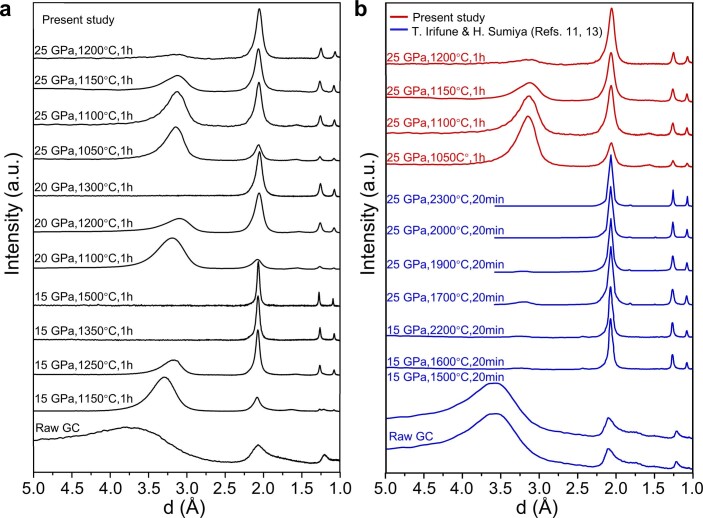
XRD patterns of samples recovered from different experimental conditions. (**a**) XRD patterns of samples we synthesized at different high-pressure and high-temperature conditions with heating duration of 1 h. (**b**) XRD patterns of samples recovered from different high-pressure and high-temperature conditions with different heating durations. The XRD patterns in blue color are from Refs. ^11,13^, and the red-colored patterns are from our present study.

Based on previous studies^10–14^ and our present experimental results, the pressure–temperature (*P*–*T*) phase diagram of GC is shown in Fig. 1c. Additional previous results on the phase transition of GC under varied pressure and temperature conditions are listed in Supplementary Table 1. The X-ray diffraction patterns of samples synthesized at 15 and 20 GPa and different temperatures are shown in Extended Data Fig. 2a, indicating the narrow temperature ranges for synthesizing the ND/DMG composite. The previous synthesis conditions close to the phase boundary of pure nano-polycrystalline diamond (NPD) or nano-crystalline diamond (NCD) in the phase diagram^13,14^ are our particular concern here. Products containing both GC (unchanged) and NCD micro-balls were obtained after treating raw GC micro-balls at 18 GPa and 1,500 °C, and at 18 GPa and 1,700 °C, with a heating duration of 1 min^14^. Furthermore, millimetre-sized bulk NPD samples with trace amounts of so-called ‘compressed graphite’ were synthesized under the conditions of 25 GPa and 1,700 °C, and of 25 GPa and 1,900 °C, for 20 min^13^. From the X-ray diffraction patterns (Extended Data Fig. 2b), the material previously synthesized at 25 GPa, 1,700 °C and 20 min resembles that synthesized under our conditions (25 GPa, 1,200 °C, 1 h). To the best of our knowledge, no detailed characterizations on microstructure or performance have been done except the determination of the grain size of NPD^13^.

**Extended Data Fig. 3:**
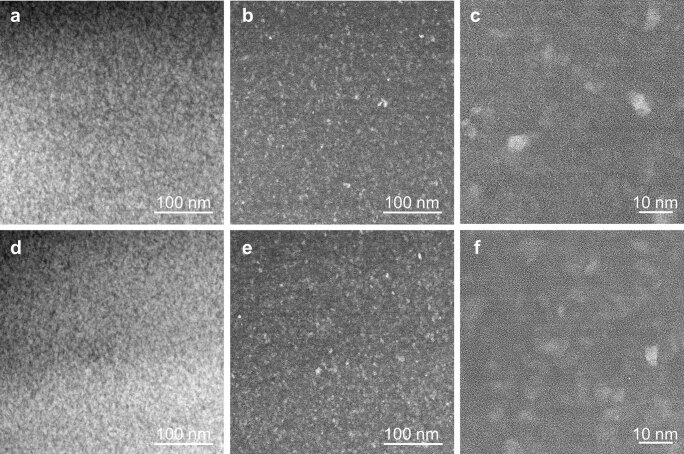
Variation of diamond content in ND/DMG composite with increasing synthesis temperature in a narrow range. (**a, b**) Low-magnification HAADF and LAADF images of Composite-1, respectively. (**c**) High-resolution LAADF image of Composite-1. (**d, e**) Low-magnification HAADF and LAADF images of Composite-3, respectively. (**f**) High-resolution LAADF image of Composite-3. Nanodiamonds (white regions) are evenly dispersed in the disordered multi-layer graphene matrix (dark regions). With the increase of synthesis temperature, more nanodiamonds appear in the composite, but no obvious grain growth is found.

**Extended Data Fig. 4:**
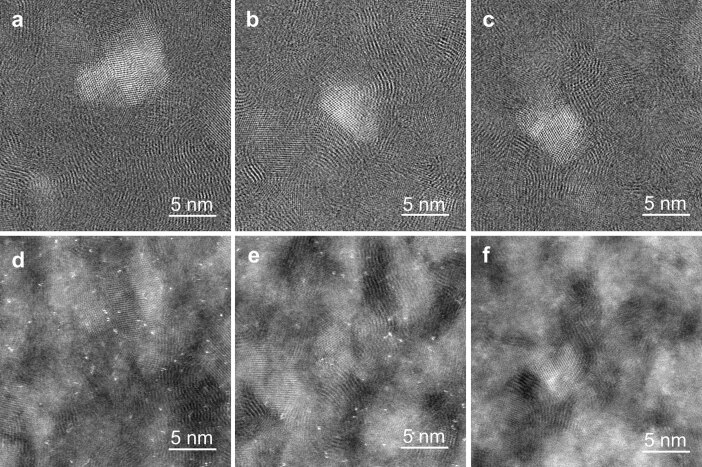
Microstructure of ND/DMG composite. (**a**-**c**) Atomic-resolution LAADF and (**d**-**f**) HAADF images of the typical microstructure of ND/DMG composite viewed along the [−101] zone axis of diamond. The dark, curved stripes with larger interlayer spacing are the disordered multi-layer graphene, and the periodic crystal lattice with white color and smaller interplanar spacing is from cubic diamond. Twins and stacking faults can be found in nanodiamonds.

The composite microstructure is further observed by scanning transmission electron microscopy (STEM), and low-/high-angle annular dark-field (LAADF/HAADF) images are shown in Extended Data Figs. 3 and 4. In the composite, the grain size of diamond crystallites ranges from 2.2 to 12.1 nm, with an average size of ~4.8 nm. All diamond crystallites are evenly embedded in the DMG matrix. With the increase of synthesis temperature, more nanodiamond crystallites appear in the composite, but no obvious grain growth is found. The HAADF-STEM observations reveal the atomic-resolution structures of diamond and disordered graphene as well as interfaces between them. The ultrafine diamond crystallites, which were generated in situ by transformation of disordered graphene fragments in parent compressed GC, are of cubic crystal structure, and the multiple sub-twins can be observed on the low-energy {111} planes of nanodiamond. The size of sub-twins in the nanodiamond grains of the composite is estimated to be ~1.4 nm, which is less than the twin thickness (~5 nm) of ultrahard twinned diamond transformed from onion-like carbon^20^.

**Fig. 2:**
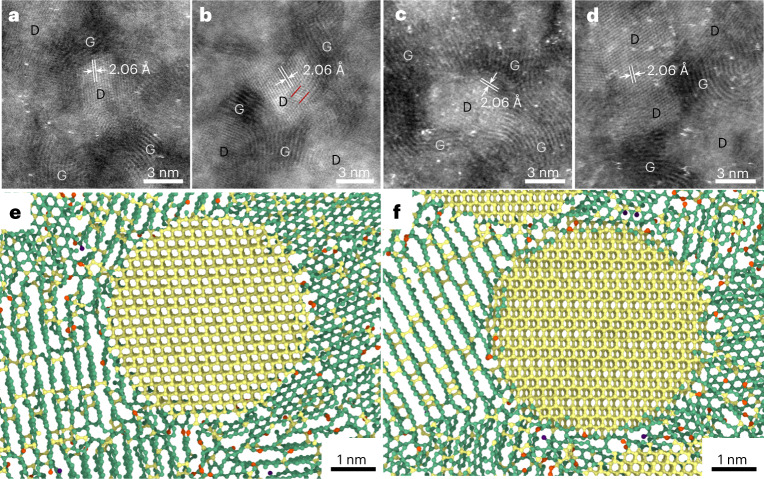
Incoherent interface structures between ND and DMG. **a**–**d**, Atomic-resolution HAADF-STEM images, revealing the complex interface structures with random, self-matching *sp*^2^ or *sp*^3^ bonding. D and G represent the regions of ND and DMG, respectively. The {111} *d* spacing of diamond is specified. Twin boundaries are indicated by red lines. **e**,**f**, Simulated atomic structure at the interface between ND and DMG. The red, green and yellow atoms are of *sp, sp*^2^ and *sp*^3^ hybridizations, respectively. The yellow nanodiamonds have a size of ~5 nm.

**Extended Data Fig. 5:**
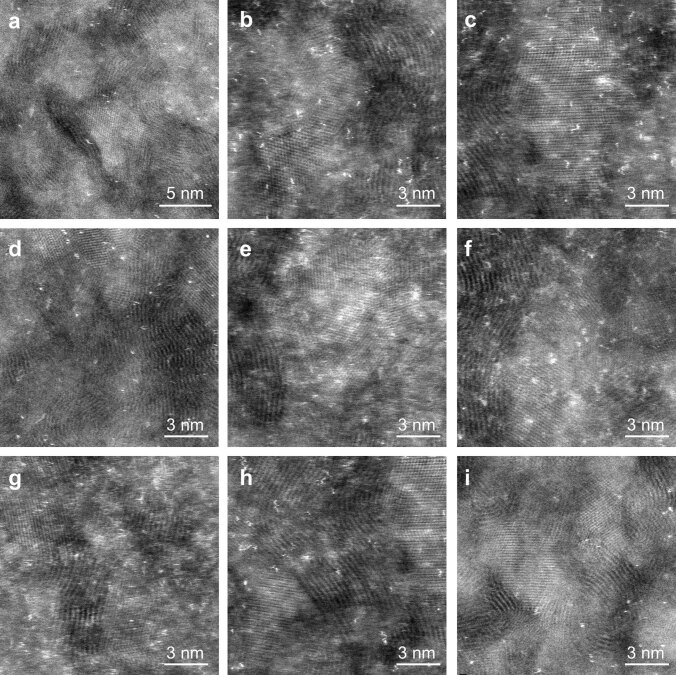
Complex incoherent interfaces between ND and DMG. (**a-i**) Atomic-resolution HAADF images of ND/DMG composite. The dark, bent fringe with larger interlayer spacing are the disordered multi-layer graphene, and the white, periodic crystal lattice with smaller interplanar spacing is from cubic diamond.

**Extended Data Fig. 6:**
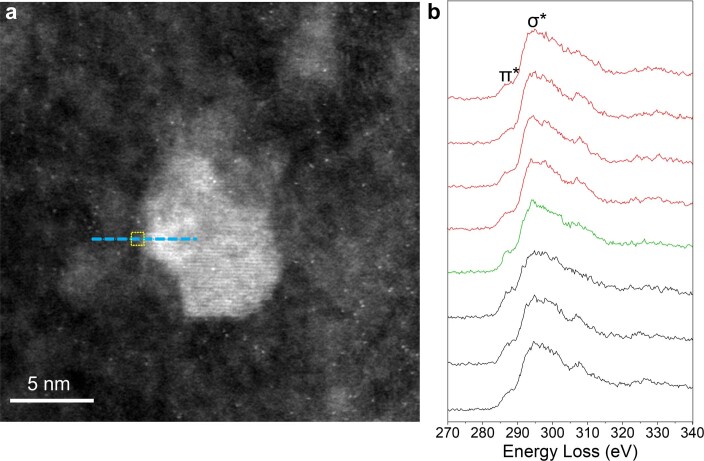
EELS line scan across the interface between ND and DMG. (**a**) Typical High-resolution HAADF image of ND/DMG composite. The deepskyblue dashed line represents the pathway of EELS line scan, and the yellow rectangle represents the interface between ND and DMG. (**b**) EELS along the blue dashed line in **a**. The spectra collected at domains of DMG, ND/DMG interface and ND were marked by black, blue and red color. There is an intensity decrease of π* peak from DMG (black spectra) to ND (red spectra), and the intensity of π* peak at the interface (green spectrum) is between those at DMG and ND.

The ND/DMG interface has irregular morphologies, some of which are polygonal and angular. As displayed in the HAADF-STEM images, the disordered graphene layers are mainly bonded to some atoms in the diamond planes at the interface through random *sp*^2^- or *sp*^3^-hybridized covalent bonds (Fig. 2 and Extended Data Figs. 4 and 5). These observations reveal that the incoherent interface between ND and DMG is generated by the GC-to-diamond transition. This incoherent interface is in strong contrast to the coherent interface caused by the graphite-to-diamond transition observed by HAADF-STEM^18^, and to the semicoherent interface (type 2 diaphite) in natural impact diamonds as observed by high-resolution transmission electron microscopy^18,21,22^. It also differs from the transient interface structure (with a graphitic interlayer distance that is actually less than 2.5 Å) that is from compressing multi-walled carbon nanotube fibres^23^. The covalent bonding at the incoherent interface benefits the improvement of the hardness and strength of the composite. In addition, it is also observed that some graphene layers are nearly parallel to the interface (Fig. 2 and Extended Data Figs. 4 and 5), and thus they are connected through a van der Waals interaction, which is similar to the recently proposed interface model of type 1 diaphite^21,22^. Although this interface mode cannot enhance the hardness of the composite, the parallel multilayer graphene at the interface is beneficial to the electrical conductivity of the composite. To illustrate the change in bonding mode at the interface between ND and DMG, a linear electron energy loss spectroscopy scan with high spatial resolution (less than 1 nm) was performed (Extended Data Fig. 6). From the DMG to ND domain, there is an obvious drop in the intensity of the π* peak; that is, the intensity of π* peak for the interface is between those of DMG and ND. This indicates that an intermediate hybridization state exists at the interface, which corresponds to the atomic structure revealed by HAADF imaging and simulated results (Fig. 2 and Extended Data Figs. 4 and 5).

The formation of an incoherent interface in the ND/DMG composite is closely related to the microstructure of the initial precursor. The currently used GC precursor is a kind of poor-graphitic disordered carbon composed of short, curved graphene fragments, and has a large number of defects in structure, such as an incomplete crystalline plane, pentagonal and heptagonal rings and dangling bonds. Under high temperature and high pressure, the structural order of the disorderly curved graphene-like fragments is improved, and the small graphitic domains with few defects can be easily rearranged into the atomic array of the most thermodynamically stable cubic diamond, which would lead to the formation of small diamond nuclei. These small diamond nuclei and other multilayer graphene fragments with more defects would generate incoherent interfaces. The further growth of diamond nuclei requires bonding and breaking processes between atoms at the interface, leading to the relatively slow growth of diamond nuclei. In this scenario, the extensive nucleation of nanodiamond at the sites of small graphitic domains occurs, and the number of nanodiamond nucleates increases with synthesis temperature. When a small number of graphene-like layers remain in the composite, the diffusion process of carbon atoms at the interface begins, thereby causing the nanodiamonds to grow and merge. The current study shows that the evolution of GC into diamond is an extensive nucleation process of nanodiamond, which is followed by the final diffusion-driven growth of nanodiamond. This structural evolution is clearly different from the transformation of graphite into diamond^18^.

### Mechanical and electrical properties

**Fig. 3:**
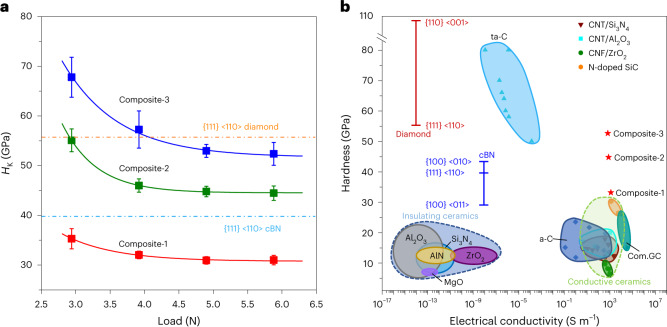
Hardness and electrical conductivity of ND/DMG composite, compared with conductive ceramics and other carbon materials. **a**, Knoop hardness (*H*_K_) of the ND/DMG composite as a function of applied loads. Error bar in **a** indicates the standard deviation (*n* = 5). The dashed lines indicate *H*_K_ of cBN and diamond crystals along the {111} <110> direction^24,25^. **b**, Room-temperature electrical conductivity-versus-hardness landscape of the ND/DMG composite and various materials. Composite-2 and Composite-3 are superhard conductive C/C composites with comprehensive properties beyond those of conductive ceramics^3–5,30,31^ and other carbon materials^24,32–36^. Except for the Knoop hardness used for the ND/DMG composite, diamond and cBN crystals^24,25^, as well as the nanoindentation hardness used for Com.GC^10^, the hardness values of the other materials in **b** are based on the Vickers scale. CNT/Si_3_N_4_, CNT/Al_2_O_3_ and CNF/ZrO_2_ refer to conductive ceramics of carbon nanotube/Si_3_N_4_, carbon nanotube/Al_2_O_3_ and carbon nanofibre/ZrO_2_ composites, respectively.

**Extended Data Fig. 7:**
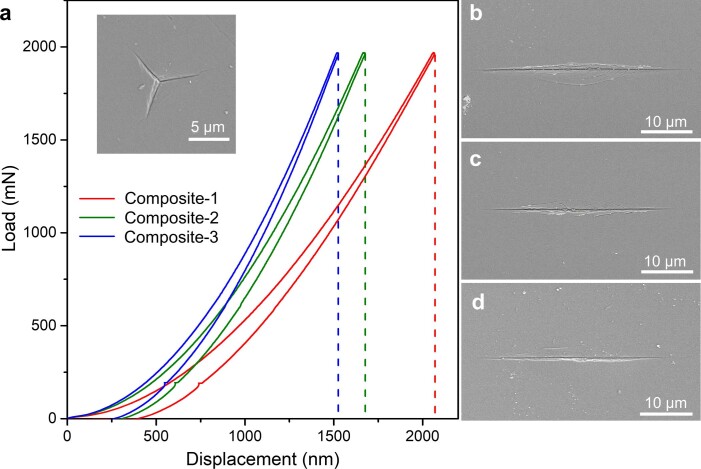
Young’s moduli and Knoop hardness of ND/DMG composite. (**a**) Load-displacement curves of ND/DMG composite with an applied load of 1.96 N. The inset shows a SEM image of a typical Berkovich indentation on the surface of Composite-3. (**b**-**d**) SEM images of Knoop indentations of Composite-1, 2 and 3, respectively. In all cases, the applied load was 4.9 N.

The Knoop hardness (*H*_K_) of the ND/DMG composite was measured by applying loads of 2.9–5.9 N, and the asymptotic hardness values of Composite-1, Composite-2 and Composite-3 are 31 ± 0.8, 45 ± 1.1 and 53 ± 1.3 GPa, respectively (Fig. 3a). Extended Data Fig. 7 shows typical scanning electron microscopy images of Knoop indentations on the polished surface of samples after a load of 4.9 N. By comparison, the *H*_K_ values of single-crystal cubic boron nitride (cBN) and diamond along the {111} <110> direction are 39 and 56 GPa, respectively^24,25^. Thus, the hardness of Composite-3 exceeds that of cBN and is comparable to that of the diamond {111} plane. Moreover, the Young’s moduli of the composite have been derived from the load–displacement curves using the Oliver–Pharr model^26^. The calculated Young’s moduli of Composite-1, -2 and -3 are 315 ± 17, 482 ± 33 and 611 ± 21 GPa, respectively (Extended Data Fig. 7).

**Extended Data Fig. 8:**
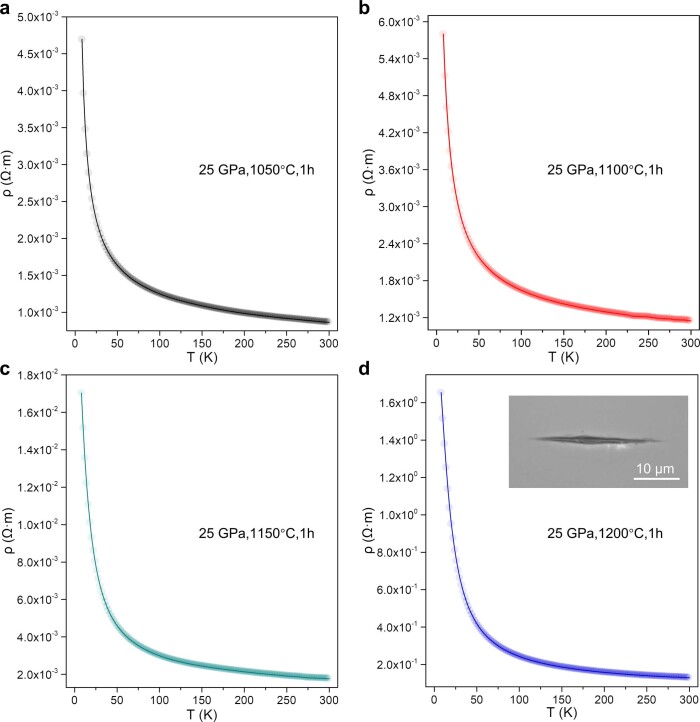
Temperature dependent electrical resistivities of samples recovered from (a) 25 GPa, 1050 °C, 1 h (Composite-1); (b) 25 GPa, 1100 °C, 1 h (Composite-2); (c) 25 GPa,1150 °C, 1 h (Composite-3); (d) 25 GPa,1200 °C, 1 h. The diamond content of these samples is ~20%, 50%, 70% and 90%, respectively. The resistivity of all the samples increases with the decrease of temperature, showing a semiconducting characteristic. The inset in (**d**) shows the optical image of Knoop indentation of the sample. The applied load is 4.9 N.

The densities of Composite-1, Composite-2 and Composite-3 are ~2.6, 2.8 and 3.1 g cm^–3^, respectively, as measured directly from the mass and cylindrical sample volume. The electrical resistivities of Composite-1, -2 and -3 are measured within the temperature range of 4–300 K by a standard four-point probe technique (Extended Data Fig. 8). The room-temperature electrical conductivity of the ND/DMG composite is in the range ~670–1,240 S m^–1^. In the composite, diamond is electrically insulating, but owing to the small average grain size of ~4.8 nm, the conductive pathways should be present in the DMG matrix and hence, facilitating the electron conduction. Considering that the nearly pure NPD material^13^ previously synthesized at 25 GPa, 1,700 °C and 20 min has a similar X-ray diffraction pattern to the material that we synthesized at 25 GPa, 1,200 °C and 1 h, we further determine the mechanical and electrical properties of our sample and find that the Knoop hardness of the sample is as high as 74 GPa, but its electrical conductivity is two orders of magnitude lower than that of Composite-3 (Extended Data Fig. 8). This implies that a slight decrease of the heating temperature in the narrow *P*–*T* range discovered in the present work causes a substantial enhancement of the electrical conductivity without lowering the hardness much. This key finding is critically important for tailoring the properties of a C/C composite and for synthesizing a high-performance C/C composite. Figure 3b shows a room-temperature electrical conductivity-versus-hardness landscape for the ND/DMG composite and various materials such as conventional insulating ceramics^27–29^, conductive ceramics^3–5,30,31^, diamond and cBN single crystals^24,25,32^, as well as amorphous carbon materials including hydrogen-free amorphous carbon (a-C)^33,34^, tetrahedral amorphous carbon films (ta-C)^35,36^ and compressed GC (Com.GC)^10^. Among the materials compared, this kind of ND/DMG composite has the advantages of super-high hardness and electrical conductivity simultaneously. It has higher hardness values than conductive ceramics, up to nearly twice that of N-doped SiC (ref. ^3^), and its conductivity is comparable to that of the best conductive ceramics^3–5,30,31^. We did theoretical analyses to verify these excellent properties of our ND/DMG composite and to further explain their structural origins (Supplementary Texts 2 and 3 and Supplementary Figs. 1 and 2).

**Fig. 4:**
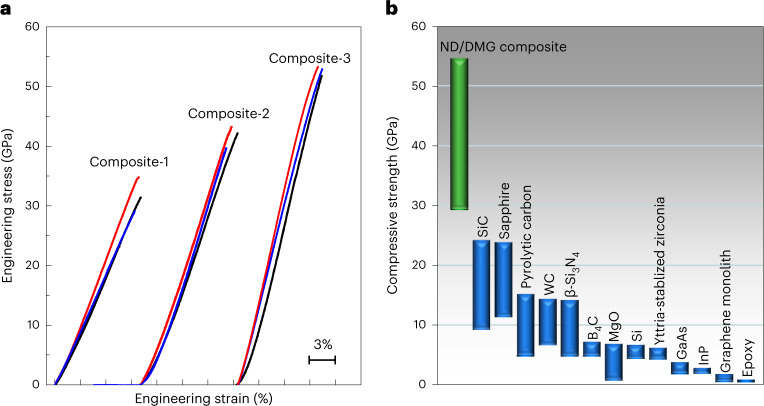
Comparison of compressive strength between ND/DMG composite and other types of materials. **a**, Typical engineering stress–strain curves of the ND/DMG composite micropillars with a diameter of ~1 μm. The micropillars can be deformed elastically up to ~10% strain and undergo catastrophic fracture at the maximum applied stress. The stress–strain curves of Composite-2 and Composite-3 are offset along the strain axis by 10% and 21.5%, respectively. **b**, Comparison of the compressive strength of the ND/DMG composite with other materials. The compressive strength values of all the reference materials^39–49^ were obtained by the uniaxial compression of micropillars. The results demonstrate that the ND/DMG composites are stronger than traditional ceramics, such as SiC, sapphire, β-Si_3_N_4_, tungsten carbide (WC), B_4_C and MgO.

**Extended Data Fig. 9:**
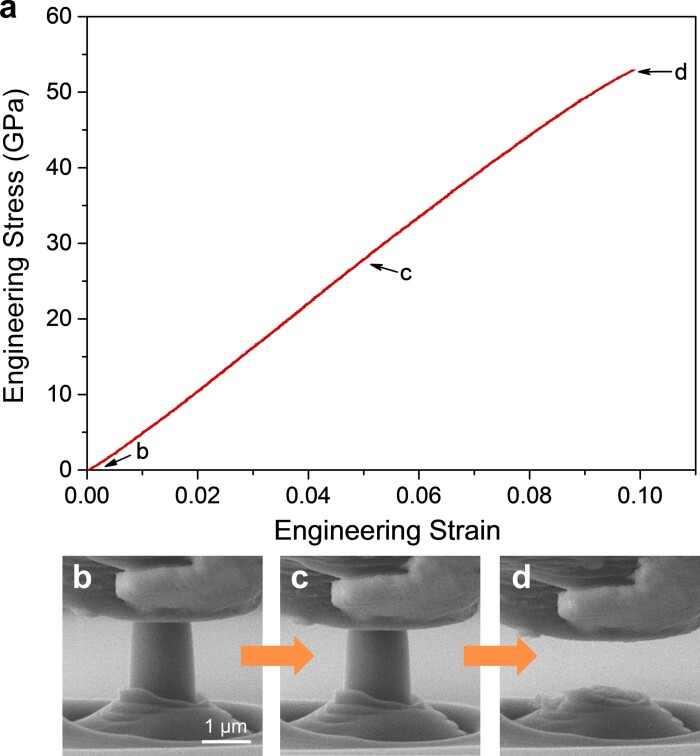
In-situ compression of Composite-3 micropillar with diameter of ~1.1 μm. (**a**) Stress-strain curve of the tested sample. (**b-d**) Deformation snapshots of the micropillar during compression, corresponding to the b-d positions in stress-strain curve. The micropillar underwent catastrophic fracture at the maximum applied stress.

The superior mechanical properties of the ND/DMG composite are further demonstrated by an in situ uniaxial compression test. Micropillars of Composite-1, Composite-2 and Composite-3 with a diameter of ~1 µm have high compressive strengths of ~28, 41 and 54 GPa, respectively (Fig. 4a). All the micropillars deform elastically until fracture occurs and have a similar compressive strain of ~10% (Fig. 4a and Extended Data Fig. 9). This large elastic strain in the ND/DMG composite exceeds those of the ‹100›-oriented and ‹111›-oriented micropillars of diamond single crystal^37^ as well as amorphous carbon micropillars^8^, and is comparable to the maximum tensile strain of [101]-oriented diamond nanobridge arrays^38^. The compressive strengths of ND/DMG composite micropillars are much higher than those of micropillars of traditional ceramic materials^39–49^ (Fig. 4b). The compressive strength of Composite-3 is more than twice that of SiC (ref. ^49^), and its mechanical properties far exceed those of a traditional C/C composite^16^. Therefore, to the best of our knowledge, Composite-3 is the hardest and strongest C/C composite. The origin of the superior mechanical properties of our C/C composite is associated with the presence of chemical bonds in the interface (leading to the creation of topological constraints^50^) and the blockage of nanodiamond domains on the propogation of shear bands. Furthermore, the uniqueness of the ND/DMG composite among carbon material systems and its comparison with diamond–metal composites and boron-doped diamond are discussed (Supplementary Text 4 and Supplementary Tables 2 and 3).

### Atomistic simulations of compression of ND/DMG composite

**Fig. 5:**
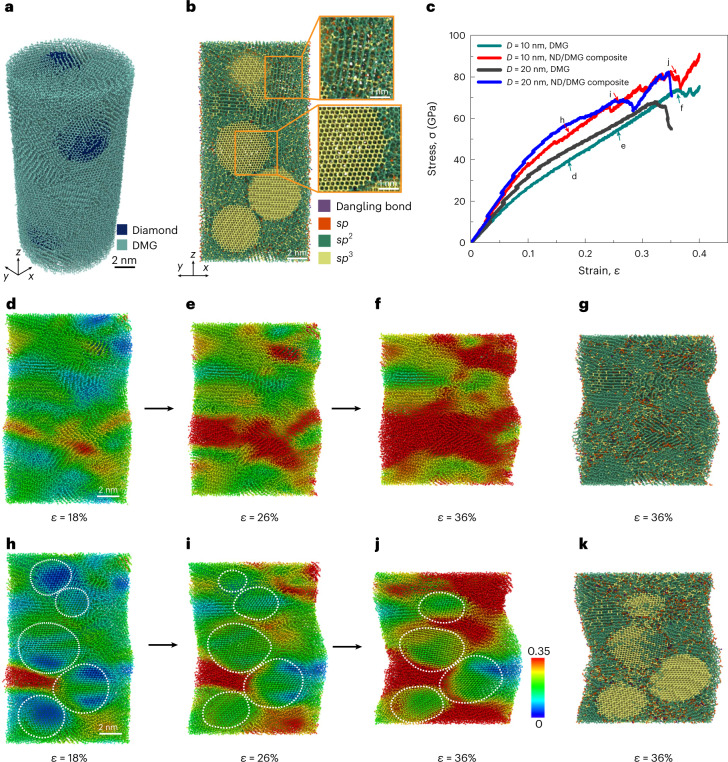
Atomistic simulations for the uniaxial compression of the ND/DMG composite and pure DMG nanopillars. **a**, Atomic configurations of the ND/DMG composite nanopillar with a diameter *D* of 10 nm. **b**, Bonding structures in the cross-section of the ND/DMG composite nanopillar with diameter *D* of 10 nm. **c**, Compressive stress–strain (*σ*–*ε*) curves of simulated samples with different diameters. **d**–**f**, A sequence of snapshots of a pure DMG nanopillar with a diameter *D* of 10 nm during compression. **g**, Bonding structures in the cross-section of the pure DMG nanopillar at a compressive strain of 36%. **h**–**j**, A sequence of snapshots of a ND/DMG nanopillar with a diameter *D* of 10 nm during compression. **k**, Bonding structures in the cross-section of the ND/DMG composite nanopillar at a compressive strain of 36%. The white dotted lines in **h**–**j** describe the profiles of diamond nanoparticles embedded in the matrix. The atoms in **d**–**f** and **h**–**j** are coloured according to their von Mises atomic strains, denoted with the colour bar. The atoms in **b**, **g** and **k** are coloured according to the bonding types.

To reveal the underlying mechanisms behind the ultrahigh strength and hardness of the ND/DMG composite, we performed a series of large-scale molecular dynamics (MD) simulations for uniaxial compression of the ND/DMG composite and pure DMG nanopillars with diameters of 10 and 20 nm. Details of the MD simulations are given in the Methods. As illustrated in Fig. 5a, the simulated ND/DMG sample consists of nanodiamonds with a diameter of 5 nm and multilayer graphene domains with an interlayer spacing of ~3.1–3.2 Å and average number of layers of 12. The simulated atomic arrangement agrees well with the LAADF/HAADF images in Fig. 2a–d and Extended Data Figs. 4 and 5. The volume fraction of diamond in the simulated composite is up to ~20.8%, which is close to that of Composite-1.

The bonding structures of the overall simulated samples are identified by calculating the coordination number of each atom (Fig. 5b). It is observed that a few *sp*^3^ bonds connect the neighbouring graphene layers, resulting in the reduced interlayer spacing in the multilayer graphene matrix, and that nanodiamonds and graphene layers are irregularly connected at the interface through mixed *sp*^2^–*sp*^3^ bonding (Figs. 2e,f and 5b). This is also consistent with the result of incoherent interface structures confirmed by LAADF/HAADF images (Fig. 2a–d and Extended Data Figs. 4 and 5). Only a small number of *sp* bonds or dangling bonds are distributed at the edges of graphene layers, on the surface of nanopillars and between nanodiamonds and graphene layers. Such hybridization among carbon atoms contributes to the high elastic modulus of the ND/DMG composite. Figure 5c presents the compressive stress–strain curves of the ND/DMG composite and pure DMG nanopillars from the MD simulations. It is evident that the ND/DMG composite has a higher elastic modulus and compressive strength than pure DMG. Undoubtedly, the higher modulus of the composite is attributed to the stronger bonding in nanodiamond, which is introduced into the composite. The higher strength of the composite should be explained in terms of the microscopic deformation mechanism.

**Extended Data Fig. 10:**
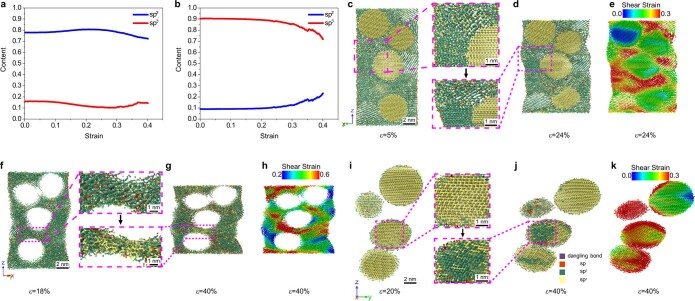
Atomistic simulations for uniaxial compression of ND/DMG composite nanopillars. (**a**) Changes of content of *sp*^2^ bonds and *sp*^3^ bonds in the multi-layer graphene matrix of ND/DMG composite nanopillar with diameter *D* of 10 nm. (**b**) Changes of content of *sp*^2^ bonds and *sp*^3^ bonds in the nanodiamond domains of ND/DMG composite nanopillar with diameter *D* of 10 nm. (**c-d**) Bonding structures in the section of ND/DMG composite nanopillar with diameter *D* of 10 nm at compressive strain of 5% and 24%, respectively. (**e**) Von Mises strain in the section of ND/DMG composite nanopillar with diameter *D* of 10 nm at compressive strain of 24%. (**f-g**) Bonding structures in the section of the multi-layer graphene matrix of ND/DMG composite nanopillar with diameter *D* of 10 nm at compressive strain of 18% and 40%, respectively. (**h**) Von Mises strain in the section of multi-layer graphene matrix of ND/DMG composite nanopillar with diameter *D* of 10 nm at compressive strain of 40%. (**i-j**) Bonding structures in the section of the nanodiamond domains of ND/DMG composite nanopillar with diameter *D* of 10 nm at compressive strain of 20% and 40%, respectively. (**k**) Von Mises stress in the section of the nanodiamond domains of ND/DMG composite nanopillar with diameter *D* of 10 nm at compressive strain of 40%. The atoms in **c-d, f-g** and **i-j** are colored according to their bonding structures. The atoms in **e, h** and **k** are colored according to their von Mises shear strains. The bond evolution in **a** is attributed to the breaking of *sp*^3^ bonds between graphene layers (**c-e**) and *sp*^2^ to *sp*^3^ (graphite-to-diamond) transformation under shear (**f-h**). The bond evolution in **b** is related to the *sp*^3^ to *sp*^2^ (diamond-to-graphite) transformation under shear (**i-k**).

Figure 5d–f and 5h–j captures a sequence of snapshots of the cross-section of compressed ND/DMG composite and pure DMG nanopillars with a diameter of 10 nm, respectively. For the pure DMG nanopillars, the shear deformation occurs in some multilayer graphene during compression (Fig. 5d). As the compressive strain increases, a local shear band forms and travels through the overall nanopillar, and is inclined to the nanopillar axis (Fig. 5e). Subsequently, the shear band gradually widens, and multiple shear bands form and intersect with each other (Fig. 5f), leading to the decrease in stress. During compression, some covalent bonds break due to the large shear, leading to the formation of more *sp* bonds and dangling bonds (Fig. 5g). However, for the ND/DMG composite nanopillar, the shear plastic deformation occurs in the DMG domains owing to their relatively weak bonding and lower strength, while the nanodiamond domains exhibit a certain shape change but do not suffer a large shear strain. Notably, diamond nanoparticles block the propagation of shear bands and further suppress the formation of shear bands (Fig. 5h–j). Such a mechanism reflects that the incoherent interface can prevent the transmission of shear strain from DMG to ND domains, and contributes to the higher compressive strength of the ND/DMG composite. During the compression of the ND/DMG composite nanopillar, the diamond structure transforms into the graphene-like structure with the increasing of *sp*^2^ bonds, indicating the graphitization of nanodiamond under high shear stress. More analyses about bond evolution are shown in Extended Data Fig. 10. Our MD simulations revealed that complex bonding occurs at the incoherent interface between the nanodiamond and DMG layers. According to the MD simulations, the higher modulus of the ND/DMG composite is attributed to the strong chemical bonds in the nanodiamond embedded in the matrix, while the higher compressive strength originates from the nanodiamond hindering the propagation of shear bands in the DMG domains.

## Conclusions

A class of ND/DMG composite has been synthesized in an optimized, narrow temperature range under elevated pressure. In the composite, nanodiamonds with an ultrafine grain size of ~4.8 nm are homogeneously embedded in a DMG matrix, and the two components are connected by random *sp*^2^ or *sp*^3^ bonding mainly through an incoherent interface. This ND/DMG all-carbon composite exhibits the synergetic effect of both diamond and disordered graphene, that is, the combination of the ultrahigh hardness/strength of diamond with the high electrical conductivity of disordered graphene. These features allow the composite to be applied as an ultrastrong conductive indenter in nanomechanics, static-free bearings and anti-static substrates and components. The current work provides a feasible pathway to synthesize a high-performance C/C composite, namely, the in situ phase transformation of metastable carbon precursors under optimum synthesis conditions.

## Methods

### Sample preparation

The raw GC rod (type-I GC, Alfa Aesar) was loaded into a hexagonal boron nitride (hBN) capsule with an inner diameter of ~1.2 mm or 2 mm and length of ~2.0 mm, and then subjected to high pressure and high temperature. High-pressure, high-temperature experiments were performed with a 10-MN double-stage large-volume multi-anvil system^51^ at Yanshan University, with the standard COMPRES 8/3 (or 10/5) sample assembly consisting of an 8 mm (or 10 mm) spinel (MgAl_2_O_4_) plus MgO octahedron with a Re heater and a LaCrO_3_ thermal insulator. Temperature was measured with type-C W–Re thermocouples, and pressure was estimated from previously obtained calibration curves at different temperatures for the multi-anvil apparatus^52^. Pressure loading/unloading rates were up to 2 GPa h^–1^. When the target pressure was reached, the sample was heated with a rate of 25 °C min^–1^ to peak temperature and then was maintained for 1 hour and finally quenched by turning off the electric power supply. Recovered samples were about 0.6–1.4 mm in diameter and 0.5–1 mm in height, and polished for further analysis.

### X-ray diffraction and Raman spectroscopy

Phase composition was identified by X-ray diffraction (Bruker D8 Discover diffractometer) with Cu Kα radiation (wavelength, *λ* = 0.15406 nm; 40 kV; 40 mA). The visible Raman spectra were measured at room temperature by using a Horiba Jobin Yvon LabRAM HR-Evolution Raman microscope with a laser radiation of 473 nm. The spot size of the laser on the sample was about 1 μm^2^.

### Transmission electron microscopy sample preparation

To eliminate grain overlaps in STEM imaging, lamellae with a thickness of ~80 nm were cut with a focused ion beam (FEI Helios 5 CX DualBeam) and further thinned to ~20 nm with low-energy Ar-ion milling (Fischione Model 1040 NanoMill). To eliminate possible carbon contamination, the lamellae were cleaned with Ar/O_2_ plasma (Gatan 695 Plasma Cleaner) for 30 s before loading into the microscope.

### HAADF-STEM and electron energy loss spectroscopy measurement

The STEM measurements were conducted with a spherical-aberration-corrected scanning transmission electron microscope (FEI Themis Z) with a monochromator operating at an acceleration voltage of 300 kV. The probe convergence angle was set at 25 mrad. The collecting angles of LAADF and HAADF were set at 16–62 mrad and 65–200 mrad, respectively. The electron energy loss spectroscopy line scan was conducted in STEM mode with an energy resolution of 0.6 eV and spatial resolution of less than ~1 nm.

### Hardness measurement

The indentation test was performed on a polished surface of samples by using a microhardness tester (KB 5 BVZ). The adopted loading time was 30 s, and a 20 s dwell time was kept at the peak load. For each sample, at least five Knoop indentations were performed at loads of 2.94, 3.92, 4.90 and 5.88 N, respectively. The Knoop hardness (*H*_K_) was determined from *H*_K_ = 14,229 *F*/*l*^2^, where *F* is the applied load and *l* is the major diagonal (longer axis) length of the Knoop indentation in micrometres. The hardness was determined from the asymptotic hardness region. The curves defining the high-load asymptotes of Knoop hardness were obtained by fitting with the Exponential Dec3 mathematical function. Young’s moduli were derived from the load–displacement curves established by the three-sided pyramidal Berkovich diamond indenter (Keysight Nano Indenter G200). The applied standard loading time to peak load was 15 s, the peak holding time was taken as 10 s and the unloading time was 15 s.

### Microcompression test

Micropillars of ND/DMG composite with a diameter of ~1 μm and diameter-to-height aspect ratio of ~1:2 were fabricated using a Ga ion beam at an acceleration voltage of 30 kV in an FEI Helios focused ion beam instrument. Initially, a current of 21 nA was used to mill craters around micropillars with diameters of ~30 μm. Then, the desired micropillar dimensions were achieved by polishing the coarse micropillars with lower currents ranging from 2,500 to 7.7 pA with concentric circle patterns, in order to minimize the damage layer. The in situ compression tests were performed in an in situ scanning electron microscopy instrument (PI-88, Hysitron) with high-loading sensors to capture in situ real-time deformation details. The experiments were conducted at a strain rate of 10^−3^ s^−1^ with a 5-μm-diameter flat punch indenter tip.

### Electrical conductivity measurement

The electrical resistivity (*ρ*) of the ND/DMG composite was measured within the range of 4–300 K with a Quantum Design PPMS-9 system by using the standard four-probe d.c. method. Platinum wires were adhered to the surface of the polished sample (~1 mm in diameter) with Leitsilber conductive silver cement (Ted Pella; silver content, 45%). The electrical conductivity *σ* is the reciprocal of resistivity, *σ* = 1/*ρ*.

### Atomistic simulations

To reveal the underlying deformation mechanisms behind the ultrahigh strength and hardness of the ND/DMG composite, we performed a series of large-scale atomistic simulations for the uniaxial compression of ND/DMG composite nanopillars with diameters of 10–20 nm via the large-scale atomic/molecular massively parallel simulator (LAMMPS) package^53^. The adaptive intermolecular reactive empirical bond order (AIREBO) force field^54^ was used to describe the interatomic interactions, including both bonded interactions and non-bonded interactions (that is, van der Waals interactions) between carbon atoms. We first constructed the simulated samples according to the microstructures of the experimental specimens. A number of layered graphite-like domains with random orientations were initially packed in two simulation boxes with dimensions of 10 × 10 × 20 nm^3^ and 20 × 20 × 40 nm^3^, and then some diamond nanoparticles were randomly inserted into some sites in these simulation boxes by replacing all the carbon atoms in corresponding sites. The constructed composite samples contained some nano-crystalline diamonds with an average diameter of 5 nm and layered graphite-like domains with an interplanar spacing of 3.1–3.2 Å and average layer number of 12. These simulated samples were first equilibrated via energy minimization and followed free relaxation for 20 ps under an isothermal–isobaric ensemble at 300 K. After equilibration, we ran a high-pressure and high-temperature process under a canonical ensemble to facilitate/accelerate the fusion of layered graphite-like domains and diamond nanoparticles. During this process, we applied a hydrostatic compression on the simulated samples at a constant strain rate of 5 × 10^8^ s^−1^ at 300 K for 62 ps. We heated the samples to increase the temperature from 300 K to 1,200 K within 10 ps, held the temperature at 1,200 K for 100 ps and finally decreased the temperature from 1,200 K to 300 K within 10 ps. We relieved the pressure of the simulated samples to zero by relaxing the samples at 300 K for 20 ps under an isothermal–isobaric ensemble. Throughout these processes, periodic boundary conditions were imposed along all three directions of the simulated sample. We extracted composite nanopillars with diameters of 10 nm and 20 nm and aspect ratios of two from the relaxed samples just described. The diamond content of the composite nanopillars was up to 20.8%, which is close to that of the experimental specimen (Composite-1). Pure DMG nanopillars with diameters of 10 nm and 20 nm were also constructed, using similar processes, and used for comparison. The composite and pure DMG nanopillars were equilibrated by free relaxation for 100 ps. During free relaxation, an isothermal–isobaric ensemble was used to maintain the temperature at 300 K and the axial stress of the nanopillars at zero. After equilibration, we applied a compression on the nanopillars along the axial direction at a constant strain rate of 5 × 10^8^ s^−1^ and a constant temperature of 300 K under a canonical ensemble. The stress of each atom was calculated based on the virial stress theorem during simulations. The axial stress of the overall nanopillar was obtained by taking the average over the axial stresses of all the atoms. Defects and bonding structure during simulations were visualized by the software OVITO (ref. ^55^).

## Online content

Any methods, additional references, Nature Portfolio reporting summaries, source data, extended data, supplementary information, acknowledgements, peer review information; details of author contributions and competing interests; and statements of data and code availability are available at 10.1038/s41563-022-01425-9.

## Supplementary information

Supplementary Information Supplementary Figs. 1 and 2, Tables 1–3 and Texts 1–4.

## Data Availability

The data that support the findings of this study are presented in the main text and the Supplementary Information, and are available from the corresponding authors upon reasonable request.
