# Supplementary material for: Ultrastrong conductive in situ composite composed of nanodiamond incoherently embedded in disordered multilayer graphene
Source: Nat Mater. 2022 Dec 15;22(1):42–9. doi: 10.1038/s41563-022-01425-9 (PMC9812777; doi:10.1038/s41563-022-01425-9)
Supplement: Supplementary file 1 — Supplementary Figs. 1 and 2, Tables 1–3 and Texts 1–4. [file 41563_2022_1425_MOESM1_ESM.pdf]

nature materials

Article

https://doi.org/10.1038/s41563-022-01425-9

Ultrastrong conductive in situ composite composed of nanodiamond incoherently embedded in disordered multilayer graphene

In the format provided by the authors and unedited

Supplementary Information

Ultrastrong conductive in-situ composite composed of nanodiamond incoherently embedded in disordered multi-layer graphene

Zihe Li[1,2†], Yujia Wang[2†], Mengdong Ma[1†], Huachun Ma[2†], Wentao Hu[1†], Xiang Zhang[1], Zewen Zhuge[1], Shuangshuang Zhang[1], Kun Luo[1,3], Yufei Gao[1], Lei Sun[1], Alexander V. Soldatov[1], Yingju Wu[1,3], Bing Liu[1], Baozhong Li[1], Pan Ying[1,3], Yang Zhang[1,3], Bo Xu[1], Julong He[1], Dongli Yu[1], Zhongyuan Liu[1], Zhisheng Zhao[1*], Yuanzheng Yue[4*], Yongjun Tian[1*] and Xiaoyan Li[2*]

[1]Center for High Pressure Science (CHiPS), State Key Laboratory of Metastable Materials Science and Technology, Yanshan University, Qinhuangdao, Hebei 066004, China

[2]Centre for Advanced Mechanics and Materials, Applied Mechanics Laboratory, Department of Engineering Mechanics, Tsinghua University, Beijing, China

[3]Key Laboratory of Microstructural Material Physics of Hebei Province, School of Science, Yanshan University, Qinhuangdao 066004, China

[4]Department of Chemistry and Bioscience, Aalborg University, DK-9220 Aalborg, Denmark

[†]These authors contributed equally to this work

*Corresponding authors: zzhao@ysu.edu.cn (ZZ); yy@bio.aau.dk (YY); fhcl@ysu.edu.cn (YT); xiaoyanlithu@tsinghua.edu.cn (XL)

Supplementary Text 1: Synthesis of the ND/DMG composite in narrow temperature range

As is well known, the sp^2-hybridized carbon forms, such as graphite, fullerenes, carbon nanotubes and GC, tend to be converted into diamond under pressure, because diamond is a thermodynamically stable phase under pressure. Theoretically, before these carbon forms are completely transformed into diamond, a diamond-containing carbon composite should be first generated in a certain synthesis temperature range. Unfortunately, this area of research has been neglected to some extent. Previous studies dealing with nanodiamond focused on synthesizing nano-polycrystalline diamond (NPD) and/or nanocrystalline diamond (NCD) with exceptional mechanical properties from GC under relatively high pressure (⩾15 GPa) and high temperature (⩾1600°C)[1-6]. Moreover, the difficulty in controlling the kinetic transition that results in the rapid formation of diamond should be another reason[1]. Specifically, GC undergoes little change under the pressure of 15 GPa at 1500°C, but it would immediately transform into nano-polycrystalline diamond when the treatment temperature was increased to 1600°C[1].

Some of the present authors demonstrated that a class of sp^2–sp^3 compressed GCs, composed of disordered graphene nano-fragments locally buckled or linked by sp^3 bonded nodes (with low sp^3 content of ~9-22%), can be obtained by isostatically compressing GC using pressure of 25 GPa at moderate temperatures of 400-1000°C[7]. The thus-derived material exhibits a remarkable combination of lightweight, electro-conductivity, high strength/hardness, and robust elastic recovery after indentation[7]. When the synthesis temperature was increased from 1000 to 1200°C at constant pressure, the compressed GC would almost completely transform into NPD[7]. The detailed pressure (P) and temperature (T) conditions and products of the previous studies[8-20] are shown in the Fig. 1C and Supplementary Table 1. This implies that the narrow synthesis temperature range of ～200°C is critical for creating the nanodiamond/amorphous carbon composite. Therefore, we chose to heat GC in the temperature range of 1000~1200°C at constant pressure to convert it into ND/DMG composite.

Supplementary Table 1 | Additional previous studies on phase transition of GC under varied pressure and temperature conditions.

**Reference**	**P/T condition**	**Transformed phase**	**Quenchable to ambient pressure (Yes/No)**
**8**	35-45 GPa/R.T.	sp^3-rich carbon phase	N
**9**	60 GPa/R.T.	sp^3-bonded carbon phase	N
**10**	33-62 GPa/R.T.	sp^3-bonded carbon phase	N
**11**	45.4 GPa/R.T.	Fully sp^3-bonded amorphous diamond	N
**12**	93 GPa/R.T.	Tetrahedral amorphous carbon	N
**13**	40-50 GPa/1800K	Amorphous diamond	Y
**14**	25-53 GPa/1800-2200K	Nanostructured diamond	Y
**15**	20 GPa/2300K	Aerogel-like diamond nanofilms	Y
**16**	100 GPa/400°C	Nanocrystalline hexagonal diamond	Y
**17**	9 GPa/2300°C<br>10 GPa/1800°C	Microcrystalline diamond	Y
**18**	5 GPa/400-1200°C<br>5 GPa/1400°C	Graphitized GC<br>Polycrystalline graphite	Y<br>Y
**19**	5.8 GPa/1375°C<br>(Ni-Fe alloy catalyst)	Graphite and diamond crystals	Y
**20**	Ambient pressure/2850-3100K	Partially graphitized GC	Y
*	25 GPa/1050-1150°C	ND/DMG composite	Y

[*] our current study. R.T. means room temperature.

Supplementary Text 2 High hardness of the ND/DMG composite

We measured the hardness of Composite-1, 2, 3, and the sample synthesized at 25 GPa, 1200°C via indentation. Their values are obtained as 31, 45, 53, and 74 GPa, respectively. The rule of mixtures (ROM) can be used to predict the effective hardness of the particle reinforced composites[21]. For the ROM, the Voigt model[22] based on the iso-strain assumption is an upper bound, while the Reuss model[23] based on the iso-stress assumption represents a lower bound. The effective hardness of composite predicted by these two models are expressed as[21]

$$H_{\mathrm{up}} = f_h H_h + f_s H_s \tag{S1}$$

$$H_{\mathrm{low}} = \left(\frac{f_h}{H_h} + \frac{f_s}{H_s} \right)^{-1} \tag{S2}$$

where H_h and H_s are the hardness values of hard and soft phases, respectively, f_h and f_l represents the volume fractions of hard and soft phases, respectively. In our ND/DMG composite, the ND is the hard phase and the DMG is the soft phase.

Supplementary Fig. 1 shows the hardness of the ND/DMG composite as a function of the volume fraction of the diamond. The upper and lower bounds predicted by Eqs. (S1) and (S2) are included in Supplementary Fig. 1 for comparison. It can be seen in Supplementary Fig. 1 that the measured hardness of the ND/DMG composite falls between the upper and lower bounds. Notably, the hardness of the ND/DMG composite with a high volume fraction (about 0.9) of the diamond gets closer to the upper bound calculated by the Voigt model (i.e. Eq. (S1)), while the hardness of composite with a low volume fraction (about 0.2) is closer to the lower bound calculated by the Reuss model (i.e. Eq. (S2)). This phenomenon is consistent with the results from previous theoretical studies about the ROM of hardness of particle reinforced composites[21]. These results indicate that the high hardness of the ND/DMG composite is related to the presence of ND.

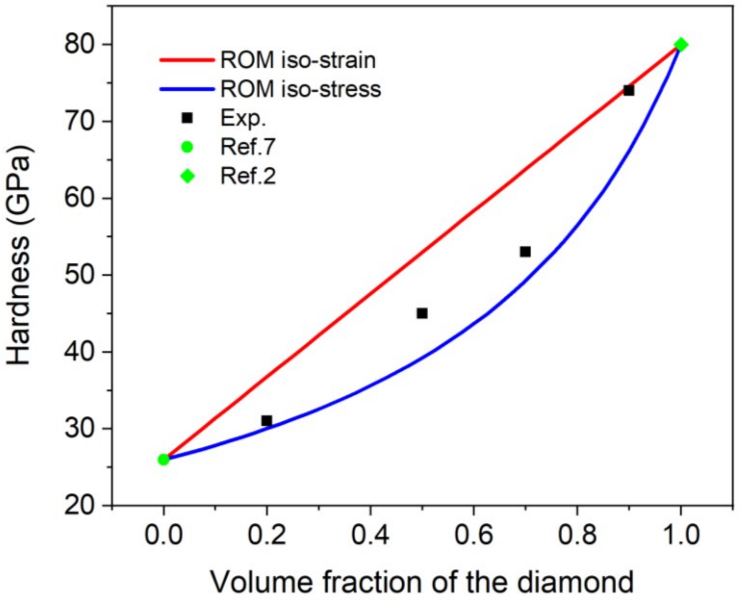

Supplementary Fig. 1 | Hardness of the ND/DMG composite as a function of the volume fraction of the diamond. The red and blue lines represent the predictions from the Voigt and Reuss models, respectively. The green data are from previous experimental measurements on the compressed glassy carbon[7] and the diamond[2].

Supplementary Text 3 High electrical conductivity of the ND/DMG composite

The room-temperature electrical conductivities of the Composite-1, 2, 3, and the sample synthesized at 25 GPa, 1200°C are measured as 1240, 769, 640, and 7.78 S/m, respectively. Various theoretical models based on the percolation theory have been proposed for predicting the effective electrical conductivities of composites consisting of high- and low-conductivity phases[24]. An improved statistical model was proposed by McLachlan[25] and can be described by the following equation,

$$\frac{f\left(\sigma_l^{1/t}-\sigma_m^{1/t}\right)}{\sigma_l^{1/t}+\left(\dfrac{1-f_c}{f_c}\right)\sigma_m^{1/t}}+\frac{\left(1-f\right)\left(\sigma_h^{1/t}-\sigma_m^{1/t}\right)}{\sigma_h^{1/t}+\left(\dfrac{1-f_c}{f_c}\right)\sigma_m^{1/t}}=0 \tag{S3}$$

where f is the volume fraction of the high-conductivity phase; σ_m, σ_h and σ_l are the electrical conductivity of the composite, the high-conductivity and low-conductivity phases, respectively; f_c is the percolation threshold, and t is the critical exponent. In the ND/DMG composite, the DMG is the high-conductivity phase, while ND is the low-conductivity phase.

Supplementary Fig. 2 shows the electrical conductivity of the ND/DMG composite as a function of the volume fraction of the DMG. The fitting curve based on the percolation model (Eq. (S3)) is included in Supplementary Fig. 2. As shown in Supplementary Fig. 2, the experimental results of electrical conductivity of the ND/DMG composite are in good agreement with the predicted values from the percolation model. The percolation threshold for the ND/DMG composite is obtained as 0.094 by fitting on the experimental data. The lowest volume fraction of DMG in our ND/DMG composite is up to 0.2, which is higher than the percolation threshold. According to the percolation theory, once the volume fraction of high-conductivity phase exceeds the percolation threshold, the composite undergoes an insulator-to-conductor transition. As the volume fraction of high-conductivity phase increases from the percolation threshold, the electrical conductivity of composite increases significantly. Therefore, our ND/DMG composite exhibits a high electrical conductivity.

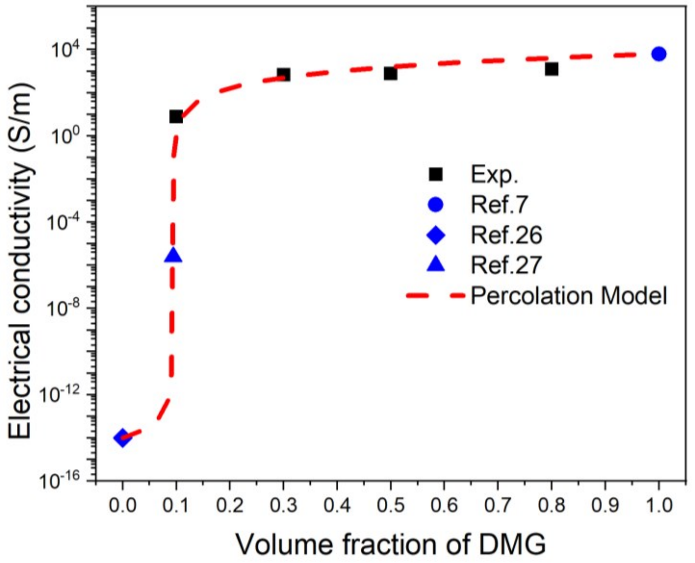

Supplementary Fig. 2 | Electrical conductivity of the ND/DMG composite as a function of the volume fraction of the DMG. The red dashed line denotes the fitting cuve based on the percolation model (Eq. (S3)). The critical exponent t and the percolation threshold f_c are obtained as 1.7 and 0.094 from fitting, respectively. The rhombus, triangular and circular data are from previous experimental measurements on the electrical conductivities of diamond[26], similar composite samples[27] quenched from 15 GPa, 2000°C and compressed glassy carbon[7], respectively.

Supplementary Text 4 Uniqueness of ND/DMG composite in carbon material system and its comparison with diamond-metal composites and boron-doped diamond

It is important to emphasize the unique position of this ND/DMG composite in carbon materials. As is well known, since elemental carbon has three hybridization modes of *sp*, sp^2 and sp^3, it possesses a variety of allotropes. Among them, graphite is an excellent conductor, and diamond is a quite good insulator at room temperature. This is because the π bonds formed by isolated electrons during the sp^2 hybridization can facilitate the electron transfer, thereby enhancing electrical conductivity while the saturated sp^3 bonds contribute to the hardness of the material, but do not benefit electron transfer. The carbon materials with sp^2-sp^3 mixed bonds are expected to exhibit the synergetic effect of graphene and diamond, i.e., both ultrahigh hardness and outstanding electrical conductivity. However, for the currently known carbon materials, ta-C with high sp^3 content is a class of superhard semiconductor films with low conductivity of 10^{-10}-10^{-6} S/m[28,29]. The hardest, strongest amorphous carbon (AM-III) has a higher sp^3 content of 94% than ta-C, and thus it should have a lower conductivity[30]. The sp^2 dominated a-C and Com.GC have the similar (or even higher) conductivity as conductive ceramics, but their hardness is less than 30 GPa[7,31,32]. The ND/DMG composite synthesized at present have a unique microstructure, in which the disordered multi-layer graphene matrix is conductive and the nano-diamond embedded in it can greatly improve the hardness of the composite, so as to finally realize the combination of conductive superhard performance in carbon materials. Supplementary Table 2 summarizes the comparisons in the synthesis conditions, phase, hardness and electrical conductivity between previous studies[30,33-39] using C_{60} as precursor and our current study. It is seen that the phase of our ND/DMG composite is distinct from those of carbon materials previously synthesized by using C_{60} as precursor. It is noted that our ND/DMG composite exhibits a combination of high hardness and electrical conductivity.

Some diamond-metal composite[40-43] and boron-doped diamond[44-47] exhibited high hardness and good electrical conductivity. In the diamond-metal composite recently studied[40-43], the metals act as the matrix, while the diamond is the reinforced phase. Thus, the strength and hardness of diamond-metal composites are lower than those of diamond materials and our ND/DMG composites. The electrical conductivity of these diamond-metal composites comes from the metal components in the composites, but the metals generally soften at high temperature. For boron-doped diamond[44-47], boron is actually difficult to be doped into diamond crystals, and its electrical conductivity is related to the content and uniformity of boron in diamond. Supplementary Table 3 shows that the electrical conductivities of boron-doped diamond single crystals[48-51] are generally lower than that of our ND/DMG composite. The conductivity of boron-doped polycrystalline diamond is mainly from the intergranular carbon-doped amorphous boron phase[45]. Such boron-rich material is prone to have chemical reaction with carbon, oxygen, nitrogen and others to form various compounds. In contrast to diamond-metal composites and boron-doped diamond, our ND/DMG composite is an all-carbon material with a combination of good electrical conductivity and high hardness/strength, which is unique in the carbon material family. Furthermore, our all-carbon ND/DMG composite has higher electrical conductivity than boron-doped diamond single crystal, better conductivity uniformity than polycrystalline boron-doped diamond and diamond-metal composites. The former is more stable than the latter under harsh chemical acid-base conditions or high temperature conditions. Certainly, the previous studies of boron-doped diamond composite provide insight into the structure-property relationship of both foreign elements-doped carbon materials and pure carbon composite. The ND/DMG composite can be used as the conductive superstrong indenters in the field of micro/nano-mechanics. These indenters can realize synchronous in-situ measurement of changes in mechanical and electrical properties of materials during compression tests, which cannot be achieved by currently commonly used single-crystalline diamond indenter. Moreover, the performance of this C/C composite material far exceeds those of the commonly used graphite and traditional C/C composites, and it is considered to be the next generation of high performance conductive superstrong mold.

Supplementary Table 2 | Comparison between previous studies using C_{60} as precursor and our study.

Reference	Synthesis condition (Pressure/Temperature)	Composition and phase	Hardness	Electrical conductivity
33	8 GPa/900K	C_{60} polymer (rhombohedral phase)	2 GPa (H_v)	<10^{-6} S/m
	8 GPa/1200K	Disordered cross-linked layered structure	30 GPa (H_v)	10^3 S/m
	8 GPa/2300K	Disordered graphite	1 GPa (H_v)	N/A
	9.5 GPa/770K	C_{60} polymer (fcc+bct phases)	80 GPa (H_v)	10^{-6} S/m
	9.5 GPa/1800K	Disordered cross-linked layered structure	25 GPa (H_v)	N/A
	13 GPa/670K	C_{60} polymer (fcc phase)	80 GPa (H_v)	10^{-2} S/m
	13 GPa/970K	C_{60} polymer (monoclinic phase)+amorphous cage nanostructure	140 GPa (H_v)	10^{-6} S/m
	13 GPa/1500K	Amorphous cage nanostructure	170 GPa (H_v)	10^{-3} S/m
	13 GPa/2100K	Amorphous cage nanostructure+diamond	N/A	10^{-2} S/m
34	20 GPa/2200°C	Aggregated diamond nanorods	N/A	N/A
35	20 GPa/2300K	Nanocrystalline cubic diamond (crystallite size: 5-12 nm)	N/A	N/A
36	18 GPa/1800°C	Nanocrystalline cubic diamond (crystallite size: 5-10 nm)	70-85 GPa (H_k, 4.9 N)	N/A
	18 GPa/2000°C	Nanocrystalline cubic diamond (crystallite size:20-100 nm)	93-104 GPa (H_k, 4.9 N)	N/A
37	20 GPa/1000 °C	Amorphous carbon (AC-1)	63.2 GPa (H_k, 4.9 N)	N/A
	27 GPa/1000°C	Amorphous carbon (AC-3)	69.3 GPa (H_k, 4.9 N)	N/A
38	30 GPa/1600K	Paracrystalline diamond	116.1 GPa (H_v, 4.9 N);	N/A
39	15 GPa/800°C	Amorphous carbon (am-I)	43 GPa (H_k, 4.9N)	5.2 S/m
	15 GPa/1000°C	Amorphous carbon (am-II)	57 GPa (H_k, 4.9N)	2.9 S/m
30	25 GPa/1000°C	Amorphous carbon (AM-I)	58 GPa (H_k, 3.92 N)	N/A
	25 GPa/1100°C	Amorphous carbon (AM-II)	62 GPa (H_k, 3.92 N)	N/A
	25 GPa/1200°C	Amorphous carbon (AM-III)	72 GPa (H_k, 3.92 N)	N/A
*	25 GPa/1050-1150°C	ND/DMG composite	31–53 GPa (H_k, 5.88 N)	670-1240 S/m

* our current study. H_v represents the Vickers hardness, while H_k represents the Knoop hardness. It is noted that the disordered cross-linked layered structure fabricated under 8 GPa and 1200K contains a high concentration of sp^2 bonds and a low concentration of randomly distributed sp^3 bonds, and its hardness and electrical conductivity are close to but slightly lower than those of our Composite-1. Its hardness is significantly lower than those of our Composite-2 and Composite-3, and is not within the superhard regime.

Supplementary Table 3 | Crystal plane, boron doped content and electrical conductivity of boron-doped diamond single crystals.

Reference	Crystal Plane	Boron Content (cm^{-3})	Electrical Conductivity (S/m)
**48**	{111}	$(1.1\text{-}2.1)\times10^{20}$	14 - 439
**49**	{001}	$7.2\times10^{17}\text{-}7.1\times10^{19}$	0.8 - 5.9
	{111}	1.25×10^{21}	19
**50**	{111}	N/A	2.5×10^{-5} - 5×10^{-2}
**51**	N/A	N/A	9.9×10^{-2} - 9.2×10^{-1}

Supplementary References

1. Sumiya, H., Yusa, H., Inoue, T., Ofuji, H. & Irifune, T. Conditions and mechanism of formation of nano-polycrystalline diamonds on direct transformation from graphite and non-graphitic carbon at high pressure and temperature. *High Press. Res.* **26**, 63–69 (2006).
2. Sumiya, H. & Irifune, T. Hardness and deformation microstructures of nano-polycrystalline diamonds synthesized from various carbons under high pressure and high temperature. *J. Mater. Res.* **22**, 2345–2351 (2007).
3. Irifune, T. *et al.* Synthesis of nano-polycrystalline diamond from glassy carbon at pressures up to 25 GPa. *High Press. Res.* **40**, 96-106 (2020).
4. Dubrovinsky, L., Dubrovinskaia, N., Prakapenka, V. B. & Abakumov, A. M. Implementation of micro-ball nanodiamond anvils for high-pressure studies above 6 Mbar. *Nat. Commun.* **3**, 1163–1167 (2012).
5. Solopova, N. A., Dubrovinskaia, N. & Dubrovinsky, L. Synthesis of nanocrystalline diamond from glassy carbon balls. *J. Cryst. Growth* **412**, 54–59 (2015).
6. Dubrovinskaia, N. *et al.* Terapascal static pressure generation with ultrahigh yield strength nanodiamond. *Sci. Adv.* **2**, e1600341 (2016).
7. Hu, M. et al. Compressed glassy carbon: An ultrastrong and elastic interpenetrating graphene network. *Sci. Adv.* **3**, e1603213 (2017).
8. Shiell, T. B. *et al.* Graphitization of Glassy Carbon after Compression at Room Temperature. *Phys. Rev. Lett.* **120**, 215701 (2018).
9. Solopova, N. A., Dubrovinskaia, N. & Dubrovinsky, L. Raman spectroscopy of glassy carbon up to 60 GPa. *Appl. Phys. Lett.* **102**, 1–5 (2013).
10. Yao, M., Xiao, J., Fan, X., Liu, R. & Liu, B. Transparent, superhard amorphous carbon phase from compressing glassy carbon. *Appl. Phys. Lett.* **104**, (2014).
11. Lin, Y. *et al.* Amorphous Diamond : A High-Pressure Superhard Carbon Allotrope. *Phys. Rev. Lett.* **107**, 175504 (2011).
12. Tan, L. *et al.* High-Pressure Tetrahedral Amorphous Carbon Synthesized by Compressing Glassy Carbon at Room Temperature. *J. Phys. Chem. C* **124**, 5489–5494 (2020).
13. Zeng, Z. *et al.* Synthesis of quenchable amorphous diamond. *Nat. Commun.* **8**, 1–7 (2017).
14. Zeng, Z. *et al.* Preservation of high-pressure volatiles in nanostructured diamond capsules. *Nature* **608**, 513–517 (2022).
15. Dong, J. *et al.* Transparent aerogel-like diamond nanofilms from glassy carbon by high pressure and high temperature. *Diam. Relat. Mater.* **96**, 90–96 (2019).
16. Shiell, T. B. *et al.* Nanocrystalline hexagonal diamond formed from glassy carbon. *Sci. Rep.* **6**, 1–8 (2016).
17. Hirano, S. I., Shimono, K. & Naka, S. Diamond formation from glassy carbon under high pressure and temperature conditions. *J. Mater. Sci.* **17**, 1856–1862 (1982).
18. Jin, C. Q. *et al.* The Unusual Morphology, Structure, and Magnetic Property Evolution of Glassy Carbon upon High Pressure Treatment. *Brazilian J. Phys.* **33**, 723–728 (2003).
19. Singh, B. P., Singhal, S. K., Chopra, R. & Sharda, K. D. Behaviour of glassy carbon under high pressure and temperature in the presence of invar alloy catalyst. *J. Cryst. Growth* **254**, 342–347 (2003).
20. Lewis, J. C. & Floyd, I. J. Reorientation effects in vitreous carbon and pyrolytic graphite. *J. Mater. Sci.* **1**, 154–159 (1966).
21. Kim, H. S. On the rule of mixtures for the hardness of particle reinforced composites. *Mater. Sci. Eng. A* **289**, 30–33 (2000).
22. Voigt, W. Ueber die Beziehung zwischen den beiden Elasticitätsconstanten isotroper Körper. *Ann. Phys.* **274**,

573–587 (1889).

23. Reuss, A. Berechnung der Fließgrenze von Mischkristallen auf Grund der Plastizitätsbedingung für Einkristalle . Z. angew. *Math. Mech.* **9**, 49–58 (1929).
24. Clingerman, M. L., King, J. A., Schulz, K. H. & Meyers, J. D. Evaluation of electrical conductivity models for conductive polymer composites. *J. Appl. Polym. Sci.* **83**, 1341–1356 (2002).
25. McLachlan, D. S., Blaszkiewicz, M. & Newnham, R. E. Electrical Resistivity of Composites. *J. Am. Ceram. Soc.* **73**, 2187–2203 (1990).
26. Demazeau, G. & Gonnet, V. Ultra-hard materials: New developments in c-BN synthesis under high pressure conditions. *High Press. Res.* **12**, 301–306 (1994).
27. Luo, K. et al. Coherent interfaces govern direct transformation from graphite to diamond. *Nature* **607**, 486–491 (2022).
28. Kleinsorge, B., Ferrari, A. C., Robertson, J. & Milne, W. I. Influence of nitrogen and temperature on the deposition of tetrahedrally bonded amorphous carbon. *J. Appl. Phys*. **88**, 1149–1157 (2000).
29. Bewilogua, K. & Hofmann, D. History of diamond-like carbon films-from first experiments to worldwide applications. *Surf. Coatings Technol.* **242**, 214–225 (2014).
30. Zhang, S. *et al.* Discovery of carbon-based strongest and hardest amorphous material. *Natl. Sci. Rev.* **9**, nwab 140 (2022).
31. Cho, N. H. et al. Chemical structure and physical properties of diamond-like amorphous carbon films prepared by magnetron sputtering. *J. Mater. Res.* **5**, 2543–2554 (1990).
32. Savvides, N. & Window, B. Diamondlike amorphous carbon films prepared by magnetron sputtering of graphite. *J. Vac. Sci. Technol. A Vacuum, Surfaces, Film.* **3**, 2386–2390 (1985).
33. Blank, V. D. *et al.* Structures and physical properties of superhard and ultrahard 3D polymerized fullerites created from solid C_{60} by high pressure high temperature treatment. *Carbon* **36**, 665–670 (1998).
34. Dubrovinskaia, N., Dubrovinsky, L., Crichton, W., Langenhorst, F. & Richter, A. Aggregated diamond nanorods, the densest and least compressible form of carbon. *Appl. Phys. Lett.* **87**, 1–4 (2005).
35. Dubrovinskaia, N., Dubrovinsky, L., Langenhorst, F., Jacobsen, S. & Liebske, C. Nanocrystalline diamond synthesized from C_{60}. *Diam. Relat. Mater.* **14**, 16–22 (2005).
36. Sumiya, H. & Irifune, T. Hardness and deformation microstructures of nano-polycrystalline diamonds synthesized from various carbons under high pressure and high temperature. *J. Mater. Res.* **22**, 2345–2351 (2007).
37. Shang, Y. *et al.* Ultrahard bulk amorphous carbon from collapsed fullerene. *Nature* **599**, 599–604 (2021).
38. Tang, H. *et al.* Synthesis of paracrystalline diamond. *Nature* **599**, 605–610 (2021).
39. Zhang, S. *et al.* amorphous carbon with high toughness , derived from C_{60} fullerene amorphous carbon with high toughness. *Cell Rep. Phys. Sci*. **2**, 100575 (2021).
40. Johnson, W. B. & Sonuparlak, B. Diamond/Al metal matrix composites formed by the pressureless metal infiltration process. *J. Mater. Res.* **8**, 1169–1173 (1993).
41. Twomey, B., Breen, A., Byrne, G., Hynes, A. & Dowling, D. P. Rapid discharge sintering of nickel-diamond metal matrix composites. *J. Mater. Process. Technol.* **211**, 1210–1216 (2011).
42. Tillmann, W. *et al.* Carbon reactivity of binder metals in diamond-metal composites-Characterization by scanning electron microscopy and X-ray diffraction. *Diam. Relat. Mater.* **38**, 118–123 (2013).
43. Yin, S. *et al.* Advanced diamond-reinforced metal matrix composites via cold spray: Properties and deposition mechanism. *Compos. Part B Eng.* **113**, 44–54 (2017).
44. Ekimov, E. A. *et al.* Superconductivity in diamond. *Nature* **428**, 542–545 (2004).
45. Dubrovinskaia, N. *et al.* An insight into what superconducts in polycrystalline boron-doped diamonds based on

investigations of microstructure. *Proc. Natl. Acad. Sci. USA* **105**, 11619–11622 (2008).
46. Luong, J. H. T., Male, K. B. & Glennon, J. D. Boron-doped diamond electrode: Synthesis, characterization, functionalization and analytical applications. *Analyst* **134**, 1965–1979 (2009).
47. Muzyka, K. *et al.* Boron-doped diamond: Current progress and challenges in view of electroanalytical applications. *Anal. Methods* **11**, 397–414 (2019).
48. Liu, X. *et al.* Boron–oxygen complex yields n-type surface layer in semiconducting diamond. *Proc. Natl. Acad. Sci. U. S. A.* **116**, 7703–7711 (2019).
49. Bormashov, V. S. *et al.* Electrical properties of the high quality boron-doped synthetic single-crystal diamonds grown by the temperature gradient method. *Diam. Relat. Mater.* **35**, 19–23 (2013).
50. Miao, X. *et al.* High-pressure and high-temperature treatment of N-rich B-doped diamonds. *CrystEngComm* **21**, 3961–3965 (2019).
51. Hu, M. *et al.* Synthesis and characterization of boron and nitrogen co-doped diamond crystals under high pressure and high temperature conditions. *CrystEngComm* **19**, 4571–4575 (2017).
